# repo: an R package for data-centered management of bioinformatic pipelines

**DOI:** 10.1186/s12859-017-1510-6

**Published:** 2017-02-16

**Authors:** Francesco Napolitano

**Affiliations:** Telethon Institute of Genetics and Medicine (TIGEM), Via Campi Flegrei 34, Pozzuoli (NA), 80078 Italy

**Keywords:** Reproducible research, R language, Data flows, Data pipelines

## Abstract

**Background:**

Reproducibility in Data Analysis research has long been a significant concern, particularly in the areas of Bioinformatics and Computational Biology. Towards the aim of developing reproducible and reusable processes, Data Analysis management tools can help giving structure and coherence to complex data flows. Nonetheless, improved software quality comes at the cost of additional design and planning effort, which may become impractical in rapidly changing development environments. I propose that an adjustment of focus from processes to data in the management of Bioinformatic pipelines may help improving reproducibility with minimal impact on preexisting development practices.

**Results:**

In this paper I introduce the repo *R* package for bioinformatic analysis management. The tool supports a data-centered philosophy that aims at improving analysis reproducibility and reusability with minimal design overhead. The core of repo lies in its support for easy data storage, retrieval, distribution and annotation. In repo the data analysis flow is derived a posteriori from dependency annotations.

**Conclusions:**

The repo package constitutes an unobtrusive data and flow management extension of the *R* statistical language. Its adoption, together with good development practices, can help improving data analysis management, sharing and reproducibility, especially in the fields of Bioinformatics and Computational Biology.

## Background

Reproducibility has been often pointed out in literature as a fundamental point in Data Analysis research. Nonetheless it has not yet received due attention in practice, particularly in the areas of Bioinformatics and Computational Biology [1–3]. The complexity of bioinformatic data and processes and the rapidly changing environments in which they are often dealt with tend to have a negative impact on best programming practices [4], which dictate careful planning, accurate design and detailed documentation.

Data flow management is an important part of Data Analysis with respect to both reusability and reproducibility [3]. Once a number of recurrent procedures are established, each of them can be encapsulated into a module. Different analysis pipelines can then be designed by properly interconnecting predefined modules. This approach elegantly fits a number of data analysis contexts in which standard procedures are combined together to build complex pipelines [5]. In addition, although with varying degrees of flexibility [6], pipeline management tools often provide the possibility of modifying existing modules or defining new ones. Besides pipeline modules, resulting data may be reused as input to other analyses, thus also requiring proper management.

Many data flow management tools have been developed with diverse features and approaches, ranging from simple command line scripting tools like Bpipe [7] to highly visual and interactive software like Galaxy [8]. Other tools are designed to add pipeline management support to specific programming languages, like Ruffus [9] and Pyleaf [4] for Python. See [6] for a recent review encompassing the whole spectrum of pipeline management tools.

The support for formalization of an analysis pipeline design is of course a precious resource in order to foster reproducibility in Bioinformatics and Computational Biology. However, it is not always rigorously applicable in practice. When developing innovative methods for a specific application, new knowledge gathered from partial results may induce a feedback loop between data and processes, with the latter being modified as a consequence of the former. In Software Engineering, similar concepts are formalized in the context of prototype-based development [10]. I and colleagues previously pointed out that an incremental development approach cycling between results and processes is often implicitly or explicitly adopted in bioinformatic research [4]. In such cases, the use of process-focused management tools may introduce unjustified overhead.

**Fig. 1:**
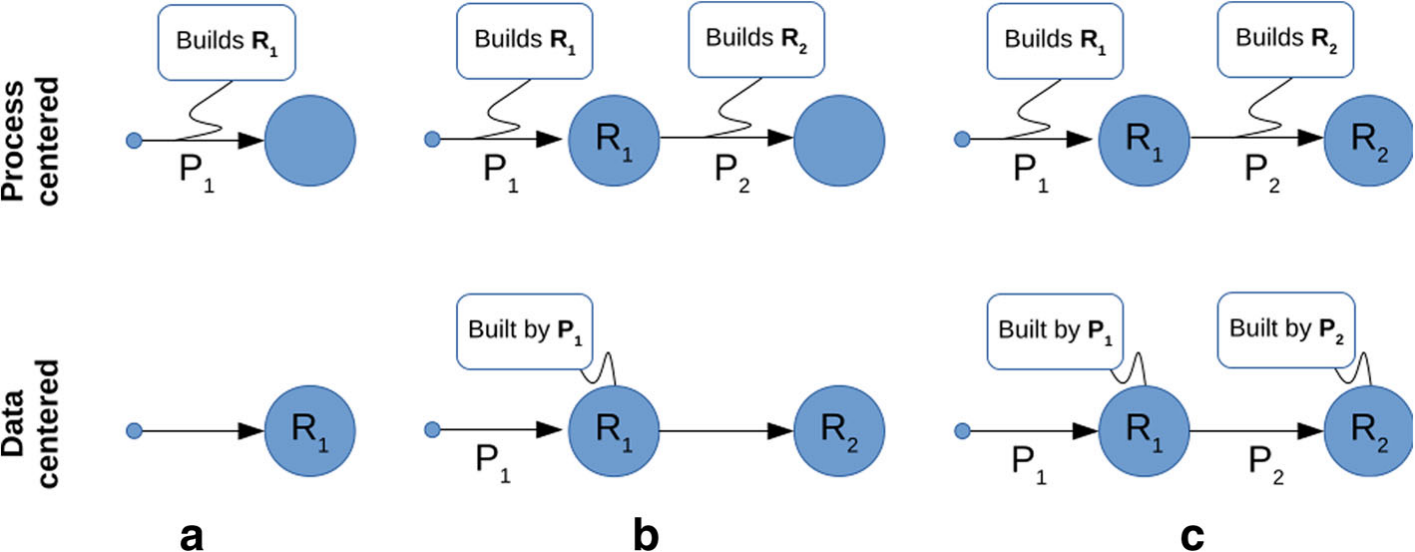
Process-centered and Data-centered approaches **a** In a process-centered approach (PCA) to the development of an analysis pipeline a well established process *P*_1_ is selected to build a new resource from input data. In the data-centered approach (DCA), the resource *R*_1_ has been created by prototypical code which needs to be properly structured and polished. **b** In the PCA the resource *R*_1_ has been produced and a well established procedure *P*_2_ has been selected to further process it. In the DCA the process *P*_1_ is now properly structured and the resource *R*_2_ has been created using prototypical code. **c** Both approaches finally yield equivalent pipelines and annotations

Figure 1 shows an ideal comparison between pure Process-Centered and Data-Centered pipeline development Approaches (PCA and DCA respectively) as defined in this article. PCA focuses on the selection or adaptation of well defined, existing processes for each stage of the pipeline. DCA relies on results obtained through prototypical methods in order to refine the processes themselves. While PCA is desirable, DCA is necessary when well established processes are not available. Of course pipeline development may proceed through hybrid PC/DC approaches in practice.

In this paper a DCA is embraced. Under this paradigm, the analysis pipeline is not seen as a well defined chain of processes to run data through, but rather as an *a posteriori* reconstruction of how data was processed. A pipeline is thus mainly conceived as a documentation tool meant to improve manageability and reproducibility of results. Its level of detail and completeness is the developer’s choice, ranging from a flat description of resources to a fully structured data flow. DCA, however, primarily focuses on proper storage, retrieval, annotation and distribution of data produced by each stage of the pipeline.

To the best of my knowledge, the *R* language currently misses extensions supporting pipeline management (either data- or flow-centered). The language does feature a range of reproducibility tools, although not dealing with pipeline management. Support for Literate Programming [11] is provided by packages such as the popular Sweave [12], allowing to mix together documentation and code in order to produce self-documenting processes. However, data itself is not part of the output. The *R* package rctrack [13] was developed to fill this gap. The tool can automatically track files accessed by *R* processes and archive them for reproducibility. This approach is certainly valuable, although it focuses on making a process reproducible, without explicit support for structuring it into a pipeline or managing the produced resources for reusability.

In the following I introduce the repo extension of the *R* statistical language. repo implements the previously described data-centered approach to pipeline management. It is publicly available from the CRAN repository [14], while more up-to-date versions are maintained at GitHub [15]. The next Section introduces the general design of the tool. A more detailed description through usage examples is presented in the “Results” Section.

## Implementation

The *R* package repo has been developed with the aim of supporting a data-centered pipeline management philosophy. The tool mainly focuses on storage, retrieval and rich annotation of data. The definition of the data flow itself is part of the data annotation. The design of repo assumes centrality of data and high variability of processes.

In order to foster reproducibility, repo implements a data repository layer which takes care of managing permanent storage of both data and annotations. Basic mandatory annotations for each stored item include a name, a textual description, and a set of tags. Additional annotations include inter-item relations and generic external attachments like rich-text documents or images.

The repo interface replaces the standard save and load *R* functions for permanent storage and retrieval. The user passes objects and corresponding annotations to repo, which transparently stores them to the file system. All items and annotations for the same repository are stored within a single directory. The inclusion of data descriptors and tags overcomes the need for directory structure since repository items are retrieved basing on annotation, as opposed to location. In particular, tags are used as a generalization of the directory tree model, as they identify possibly overlapping sets of items.

Repositories in repo are self-contained by design, so that an entire repository can be easily shared. Moreover, inside the repository directory all metadata are contained within a single file, i.e. the *index*. In fact, the index file alone can be conveniently shared. It allows to browse through all items and annotations of a repository taking advantage of all repo features not dealing with actual data, such as data analysis flow visualizations. Support for remote download can be exploited to selectively obtain data of interest.

In repo the data pipeline is actually reverse-engineered from relational annotations. For example, the user may store source code file as a repository item and annotate other items as being generated by it. Special comments in the source code can be added to associate a specific code section with the production of a resource. Dependency between items can also be annotated. The tool is aware of the data flow implicitly defined by annotations and supports batch actions on interrelated items. In repo the data flow definition is thus optional as any other annotation.

repo is an *R* language extension developed using the Reference Class paradigm [16]. In the *R* environment the user creates an object of class repo associated with a file system directory and controls the corresponding repository through the object methods (see Table 1 for a summary of the available methods). In the next Section a more detailed view of the tool is provided through direct examples.

**Table 1:** A summary of commands available in the latest development version of the repo package

Command	Description
attach	Store a generic file into the repository.
attr	Retrieves item attributes.
build	Runs code chunk associated with an item and dependant items if needed.
bulkedit	Saves repository meta data to a text file for offline editing or loads the file after editing.
check	Checks MD5-consistency of stored items.
chunk	Displays the code chunk associated with an item.
copy	Copies items between repositories.
cpanel	Runs visual interface.
dependencies	Returns and/or plots item dependencies.
export	Saves the contents of a repository item to a file in RDS format.
find	Searches all metadata for a partial string match.
get	Loads an item into the current workspace.
handlers	Returns a list of functions to be used as an alternative interface to the repository.
has	Checks wether an item is present in the repository.
info	Displays a summary of information about a regular item, a project item, or the repository.
lazydo	Evaluates specified code caching results in the repository. Loads results if already cached.
options	Sets default parameters to be used by subsequent calls to the put command.
pies	Shows statistics about disk space used by each item in the repository.
print	Summarizes information about items.
project	Creates a special “project” item.
pull	Overwrites item contents by downloading data from the associated URL.
put	Stores new data into the repository.
related	Lists items related to a given item according to dependencies.
rm	Removes items from the repository.
root	Returns repository root position on the file system.
set	Updates an existing item.
stash	Stores an item with unspecified meta information.
stashclear	Removes stash-ed items.
sys	Runs a system command on a given item.
tag	Set tags for an item.
tags	Retrieves tags for an item.
untag	Removes specified tags from an item.

## Results

This Section illustrates the main features of the repo package and its philosophy through an application example. The example involves the creation and population of a repository, its exploration, manipulation and distribution.

### Repository creation and population

In repo all the data and annotations for a single repository completely reside under a specified file system position. One repository can store resources produced by different analyses. The choice between the creation of a single central repository or multiple project-specific repositories is up to the user. The following code creates a new, empty repository in a temporary directory:



The example code reported in this Section is contained in a file named article.Rnw. The next code block stores the source code as a repository item. The attach function stores generic files (as opposed to *R* objects) in the repository. An item description and a list of tags are also specified. The project command creates a special repository item containing pipeline-wise information. The options commands sets the default source file and the default project to associate items with.



This example uses the “Mice Protein Expression Data Set” from the UCI repository [17]. In the following block the data is downloaded and a copy is stored in the repository, specifying the download URL. The URL field is useful to trace the provenance of the data, but can also be used to download the item contents through the pull function. The variable xls.name which contains the name of the downloaded file, is also used to set the identifier of the newly created object in the repository.



The stored data is not in *R* format. The following code imports it into the variable data and permanently stores the variable in the repository through the put function. In this case two relations are annotated for the newly created item: the generating source code, set as the file article.Rnw; and a dependency from the downloaded file (xls.name variable). Note that Mice Cortex is annotated as being dependent on the appropriate repository item, which contains both necessary and sufficient data to build the newly created resource. However, the actual code loads the data from the downloaded file and uses a variable defined elsewhere (xls.name). These inconsistencies with the process will be fixed later in accordance to the data-centered paradigm (see Fig. 1).



The dataset includes missing values and non-real variables. As a preprocessing step, all incomplete samples are removed and a reduced version of the dataset is stored. Dependence of the reduced set from the full set (just stored as Mice Cortex) is also annotated.



Suppose that a change is decided about the data preprocessing step. One may want to overwrite the current Mice Cortex notNA item, but keeping the previous one as a possible alternative. repo implements a simple versioning system to accomplish this task. The following code creates a scaled version of the dataset and overwrites the previously created Mice Cortex notNA item. However, since the parameter replace is set to addversion, the old item is preserved with a new name, as shown by the print output.



The attach function can be exploited to store visualizations in the repository and link them to the data they represent. The following code plots a 2-dimensional visualization of the Mice Cortex data to a PDF file and attach-es it to the item containing the corresponding data (using the to parameter).



The accuracy of the 2D plot is bound to the amount of variance explained by the first two Principal Components of the reduced dataset. The following code creates a plot of the variance explained by each Principal Component and attaches it to the previous plot.



### Repository exploration

repo supports a few commands to visualize information about a repository or a set of items. Global information can be visualized through the info command as follows.



**Fig. 2:**
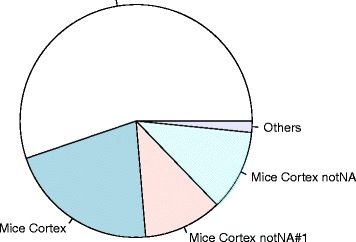
Example of repository statistics *Pie* chart visualization of the repository items according to their memory usage on the disk, as produced by the pies function



It is also possible to visualize the composition of the repository in terms of memory usage through the pies function (see Fig. 2).

Other details about single items can be visualized using the print function. Some items (like attachments) are *hidden* by default. The code below lists all the items in the repository, including hidden ones.



**Fig. 3:**
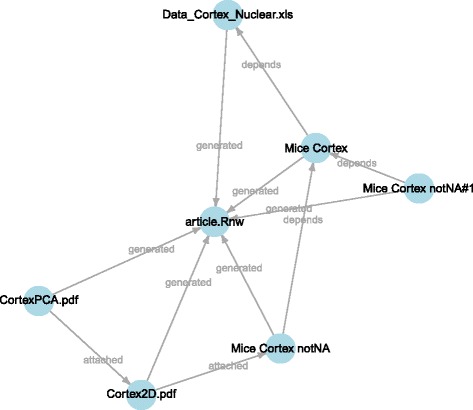
The dependency graph summarizing relations between items in the repository. Three types of relations are supported by repo: *attached to*, *depends on*, *generated by*. When items are properly annotated, this visualization also represents the analysis data flow



Three types of relations between items are supported in repo: *attached to*, *depends on*, *generated by*. Such relations can be represented through a directed graph. The dependencies function creates the corresponding visualization (see Fig. 3). When items are properly annotated, such visualization defines the analysis *data flow*.

**Fig. 4:**
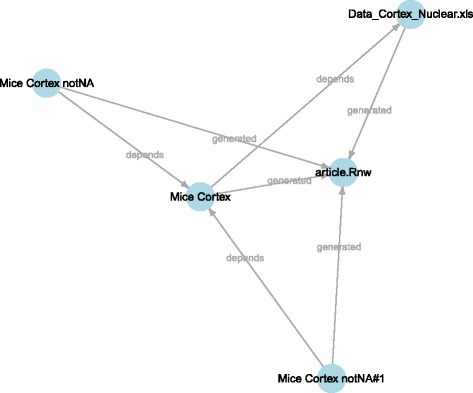
Selective plot of dependencies within the repository. In this case all the items annotated with the tag “visualization” are excluded



As a repository grows, it may contain a large number of items from multiple projects. In order to properly identify item subgroups, *tags* can be exploited as filters. Tags are supported by many repo functions and can be combined using different logic operators. In the next code block the plot items (associated with the tag “visualization”) are excluded from the dependency graph (see Fig. 4).

**Fig. 5:**
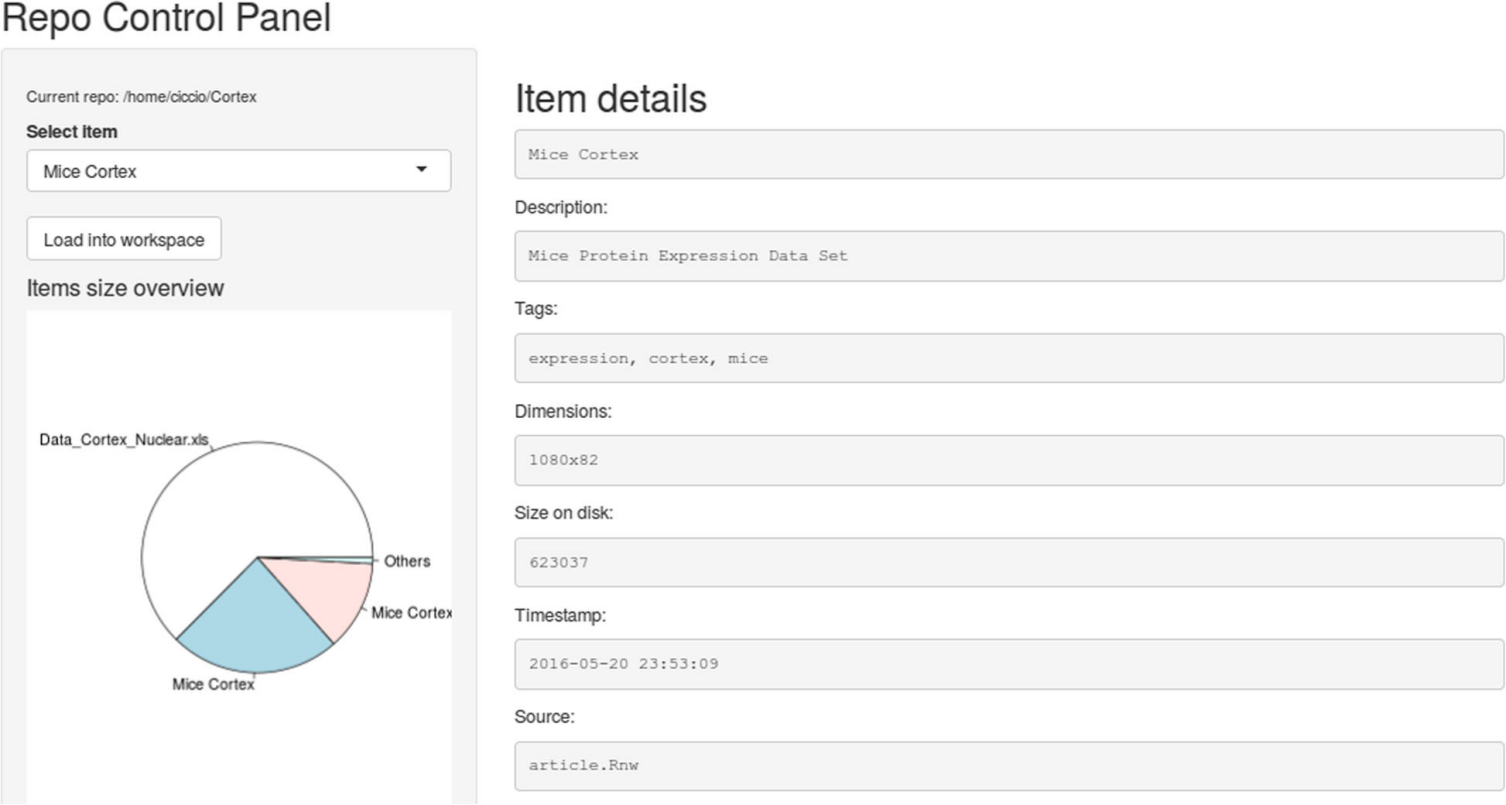
The repository control panel. It is constituted by a Shiny [20] application running in an Internet browser. The user can browse through repository items and load them into the current workspace



The repo package also includes a preliminary visual interface (see Fig. 5). The current version allows to browse repository items and load them into the current workspace.

### Items access

The most used command in repo is get. get loads an item from the permanent storage basing on its name.





On the other hand, all the details stored for a single item are reported by the info function. The summary also reports the dimensions of the data, its creation date, the storage space used, the relative file system path to the file containing the data, and an *MD5 checksum*.



If the exact identifier is unknown the find function can be used to perform a string matching against all item details.



### Analysis reproducibility

While repo focuses on data, it also supports features directly dealing with processes. Such features make the tool able to reproduce resources basing on the code they were annotated to. Reproducibility is also supported by the special project items, which collect information about an entire analysis, including the list of resources involved, R version used and necessary libraries. The info command implements a special behaviour for project items, as shown in the following:



Items in the example repository have dependencies set, thus enabling to trace back which data were used to build each resource. This may provide significant help in reproducing an analysis or reuse produced items in other analyses. However, the exact process building each resource is not described, as a generic source file is associated with all of them. Following the data-centered approach (see Fig. 1), once the analysis is well assessed, source code can be cleaned up and single processes assigned to each item. Although the code used for this example is rather simple, the following is a refinement of the block related to the Mice Cortex resource:



Note that the xls.name variable is not used anymore, and the downloaded data set is loaded from within the repository. This code is now both necessary *end* sufficient to build the Mice Cortex resource if its dependencies are satisfied. The comments starting with *“## chunk”* will be used by repo to associate the Mice Cortex resource with the actual instructions that are necessary to build it. The following lines update the source code of the project by resetting its content and show the newly defined code chunk:



The build command runs the code associated with a resource. By default, if the resource has dependencies not already present in the repository, their associated code is run first, recursively. Otherwise their code chunks are skipped. It is also possible to set a session-wise option to determine other behaviours. For example, the following code can be used to download the latest version of the file “Data_Cortex_Nuclear.xls” and build the corresponding Mice Cortex object, without overwriting the respective previous versions. Annotation of the Data_Cortex_Nuclear.xls code chunk, as shown above for the Mice Cortex chunk, is assumed.



As previously explained, when new versions of existing items are created, the latter are renamed by adding an incremental version number. Note that, thanks to the mechanism of code chunk annotation, repo supports reentrancy [6] at each properly defined pipeline stage.

### Data exchange

The repo system stores data and metadata into subfolders of the repository root in the *R* standard *RDS* format. Internally, all references to stored files are relative to the root directory, implying that each repository is completely self-contained and can be easily cloned or moved. Dedicated support for data exchange is described in this Subsection.

The tool can handle multiple repositories and copy items from one repository to another. For example, the code below creates a new repository and copies two items to it:



The related function returns the names of all items that are directly or indirectly linked to a given item, thus allowing to select an independent set of items. In the following such a set is saved to the standard *R* data format *RDS* (or their original format for *attachments*) using the export function.



An interesting application of the URL annotation regards the distribution of repositories. The buildURL parameter of the set function can be used to assign a base URL to all items. The code below copies the previously selected set of items to the repository rp2 and sets a base URL for all items (except Data_Cortex_Nuclear.xls).



Once the repository directory is copied to a public website, its index (i.e. the file R_repo.RDS in the repository root) can be distributed. Users can then selectively download items of interest using the pull repo function. The check command can be used to run an integrity check on all repository items.



## Discussion

The “Results” Section shows how the repo package can be used in the usual context of *R* development by replacing the common actions of storing and retrieving processed data with feature-reach calls to a data abstraction layer. A summary of the described commands together with other currently supported commands is reported in Table 1. Dependency annotations are used by the tool to reconstruct the data flow, and exploit such implicit structure both for data management and documentation purposes. The tool does not require any particular structuring of the code into modules or any coding conventions in general, allowing the developer to use his preferred programming paradigm and framework. However, resources can be easily associated with any portion of consecutive lines of code in order to define the exact process associated with a pipeline stage. repo features for data management and annotations are now well established and included in the stable version available on CRAN [14]. The complementary process management features, such as the chunk and build commands, instead, are included in the latest version of the package [15] and constitute the current development focus of repo. Proper storage of resources and processes can greatly help in making data pipelines manageable and reproducible, within the same lab or across different labs. However, repo currently misses support for standard data exchange formats, which limits reproducibility of data flows across platforms [18, 19], posing a stimulating priority for further development of the tool. Finally, the support of most repo features through its visual interface will improve its overall usability, particularly for inexperienced users.

## Conclusions

Data Analysis management tools can greatly help in making computational research manageable and reproducible. However, in rapidly changing development environments the implied overhead may constitute a significant obstacle. I developed the repo *R* package for data-centered pipeline management with the aim of supporting reproducible analysis while keeping design and documentation overhead at a minimum. This is achieved by supporting the management of data and metadata storage and retrieval within the *R* environment. Future developments of repo include the support for data exchange formats and coverage of most features through the visual interface. The tool is publicly available from the CRAN repository [14]. More up-to-date versions are maintained on the GitHub web site [15].

## Availability and requirements

**Project name**: repo

**Project home page**: https://github.com/franapoli/repo

**Archived version**: 10.5281/zenodo.159584

**Operating system(s)**: Platform independent

**Programming language**: R

**Other requirements**: R environment including digest and tools packages. Tested on R version 3.2.3.

**License**: GNU GPL

**Any restrictions to use by non-academics**: no restrictions

## Abbreviations

CRAN: Comprehensive R archive network
DCA: Data centered pipeline development approach
PCA: Process centered pipeline development approach
